# Association between Autism Spectrum Disorder, Trace Elements, and Intracranial Fluid Spaces

**DOI:** 10.3390/ijms25158050

**Published:** 2024-07-24

**Authors:** Matej Mlinarič, Maja Jekovec Vrhovšek, David Neubauer, Alenka France Štiglic, Joško Osredkar

**Affiliations:** 1 Department of Endocrinology, Diabetes and Metabolic Diseases, Division of Paediatrics University Medical Centre Ljubljana, Zaloška c. 2, 1000 Ljubljana, Slovenia; 2 Department of Child, Adolescent and Developmental Neurology, Division of Paediatrics University Medical Centre Ljubljana, Zaloška c. 2, 1000 Ljubljana, Slovenia; 3 Department of Child, Adolescent and Developmental Neurology, University Medical Centre Ljubljana, Bohoričeva 20, 1000 Ljubljana, Slovenia; 4 Clinical Institute of Clinical Chemistry and Biochemistry, University Medical Centre Ljubljana, Zaloška cesta 2, 1000 Ljubljana, Slovenia; 5 Faculty of Pharmacy, University of Ljubljana, Aškerčeva 7, 1000 Ljubljana, Slovenia

**Keywords:** autism spectrum disorders, trace elements, ventricular indexes

## Abstract

(1) Autism spectrum disorder (ASD) belongs to the group of complex developmental disorders. Novel studies have suggested that genetic and environmental factors equally affect the risk of ASD. Identification of environmental factors involved in the development of ASD is therefore crucial for a better understanding of its etiology. Whether there is a causal link between trace elements, brain magnetic resonance imaging (MRI), and ASD remains a matter of debate and requires further studies. (2) In the prospective part of the study, we included 194 children, including an age-matched control group; in the retrospective study, 28 children with available MRI imaging were included. All children had urine analysis of trace elements performed. In those with available brain MRI, linear indexes for the ventricular volumes were measured and calculated. (3) We found the highest vanadium, rubidium, thallium, and silver levels in children with ASD. These elements also correlated with the estimated ventricular volume based on MRI indexes in children with ASD in the subanalysis. However, the severity of the deficits did not correlate with brain MRI indexes of our elements, except negatively with magnesium. (4) Trace elements have an impact on children with ASD, but further multi-centric studies are needed to explain the pathophysiological mechanisms.

## 1. Introduction

Autism spectrum disorder (ASD) belongs to the group of complex developmental disorders. In ASD communication, behavior and symptoms that include persistent deficits in social communication, social interaction, and repetitive, restricted patterns of behavior, interests, and activities are present [1]. Epileptiform abnormalities are commonly reported among children with ASD, with frequencies ranging from 10% to 72% [2]. The cause of ASD is still not known and it is thought to be a combination of genetic and environmental factors, which equally affect the risk of ASD. However, only 15–30% of these problems can be explained by genetics [1]. Identification of environmental factors involved in the development of ASD is therefore crucial for a better understanding of its etiology. There is increasing evidence of gut microbiota–brain axis involvement in ASD, related to gastrointestinal dysfunction manifested by increased intestinal permeability and dysbiosis of gut microbiota [3]. Additionally, there is increasing evidence of the impact of exposure to toxic elements and deficiency of essential minerals on the involvement in ASD [4,5,6,7].

Trace elements play an essential role in the central nervous system. Their deficiency or excess can cause neurological diseases, including ASD. Some trace elements, such as Mn, Zn, Cu, Se, and Fe, are required by the human body for the normal functioning of all organs. On the other hand, trace elements such as Hg, Pb, As, and Al are not necessary for the normal functioning of the human body. Their accumulation in the body is detrimental to health and has no positive effects on the body [8]. They can enter the body in different ways: through the skin, inhalation, or consuming contaminated drinking water and food. Prolonged exposure to toxic elements can cause an imbalance in the body as they replace essential elements. Trace toxic elements accumulate in human body organs such as the brain, heart, liver, and kidneys, destroying normal biological functions and causing an imbalance of antioxidants. Whatever molecular pathways they affect eventually produce reactive oxygen species (ROS) that can cause oxidative stress (OS) [4].

Like any other agent humans encounter in the environment, trace elements are present in different forms and concentrations. People may react to this exposure in different ways, depending on whether these agents are toxic or essential for normal physiological functioning. The Supplementary Materials file contains the toxicological profiles of trace elements and compiles toxicological profiles of trace elements based on the source of exposure, its effect on health, and regulatory recommendations.

Understanding the impact of environmental factors on the development of ASD is essential, as these factors can be influenced. In particular, the burden of toxic elements such as Hg, Pb, As, and Cd and the deficiency of essential minerals such as Cu, Se, and Zn in the human body affect children’s health [9].

Structural magnetic resonance imaging (MRI) conducted in young children with ASD assessed the presence of brain volume overgrowth, an aberrant pattern of cortical thickness, and increased cerebrospinal fluid volume in the subarachnoid space. Moreover, connectivity studies constantly report a disruption of brain connectivity in toddlers with ASD [2,10]. Brain MRIs frequently detect incidental findings (IFs) in children with ASD. IFs are “unsought asymptomatic brain findings generated while seeking other information of interest” [2].

Whether there is a causal link between trace elements, brain MRI, and ASD remains a matter of debate and requires further studies.

## 2. Results

### 2.1. Data on the Study Groups

A total of 194 children were included in the prospective study, 43 (22.1%) were female. The average age of all children was 9.71 ± 3.62 years. The number of children in group 1 with ASD was 107; of them, 13 (12.1%) were female, and their average age was 9.16 ± 3.28 years. The number of children in group 2 with high-functioning ASD (HF-ASD) was 38. Of them, 5 (13.2%) were female, and their average age was 12.02 ± 3.19 years. The number of children in group 3—the healthy control group—was 49. Of them, 25 (51%) were female, and their average age was 9.14 ± 3.98. In the retrospective study, anonymized data from 28 children with available MRI brain images were included; 3 (10.7%) were female. Their average age at laboratory evaluation was 9.85 ± 3.07 years, and their average age at MRI imaging was 8.63 ± 3.73 years. When further divided, 21 children in the retrospective study were from the previous group 1 (children with ASD). Of them, 1 (4.8%) was female. Their average age at laboratory evaluation was 9.07 ± 2.72 years, and their average age at MRI imaging was 7.87 ± 3.68 years. Seven were from group 2 (children with HF-ASD). Of them, 2 (28.6%) were female. Their average age at laboratory evaluation was 12.18 ± 3.04 years, and their average age at MRI imaging was 10.91 ± 3.07 years. None of the healthy children had an MRI brain exam performed.

### 2.2. Concentration of Trace Elements in Urine Samples

**Figure 1:**
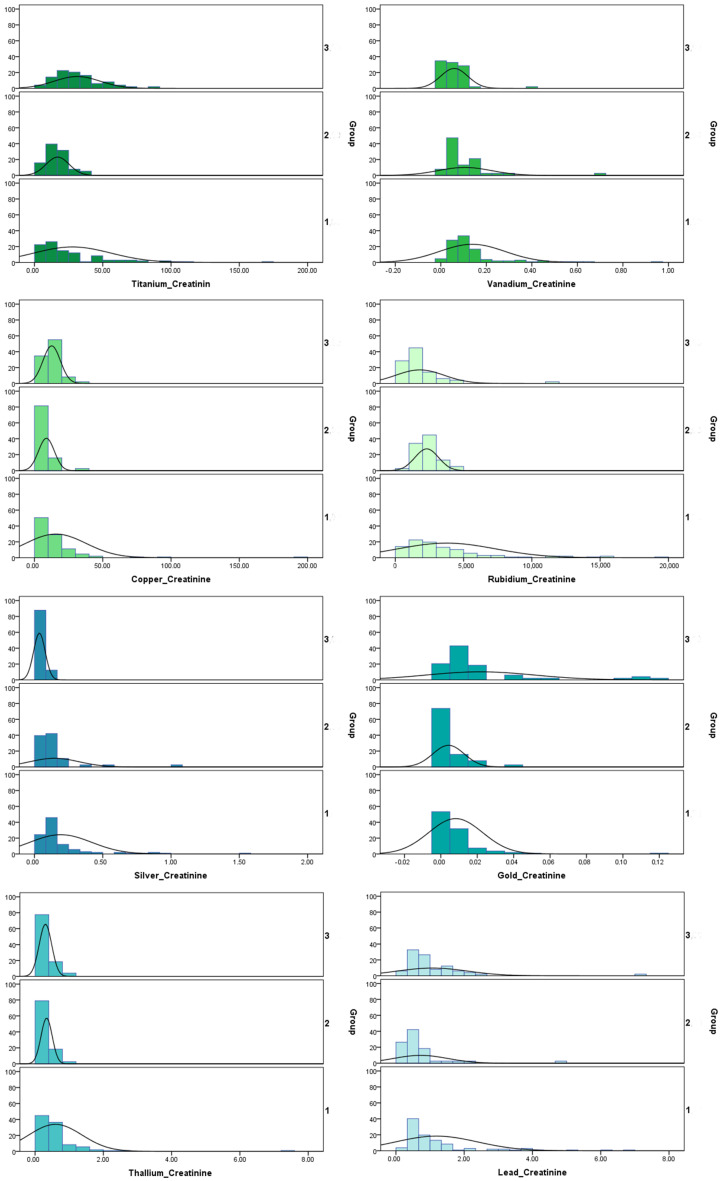
Distribution of the concentrations of trace elements per creatinine in the different groups studied. group 1: Children with autism spectrum disorder; group 2: Children with high-functioning autism; group 3: controls.

To show the concentration per creatinine of each toxin in the groups of patients with autism spectrum disorder (Group A), Asperger syndrome (Group B), and healthy controls (Group C), we calculated the median and the 25th and 75th quartiles (Table 1). Because of the non-normal distribution, we used the Whitney U test to compare the groups. The *p*-values are presented in Table 2. Because of the more significant number of tests, we added the Bonferroni factor to the *p*-values. For eight trace elements whose concentrations were statistically significantly different when compared to the other groups, the distributions are presented in Figure 1.

#### 2.2.1. Comparison of Metal Concentrations between Children with ASD and Healthy Controls

When comparing the levels of five trace metals (out of 30) in urine per creatinine between the group with ASD and the control group, statistically significant differences were observed. The levels of four trace elements were higher in the ASD group compared to the control group: vanadium (0.09 (0.06–0.15) vs. 0.05 (0.02–0.08); *p* < 0.001), rubidium (2682.9 (1581.3–4750.4) vs. 1395.11 (752.9–2113.3); *p* < 0.001), silver (0.12 (0.09–0.19) vs. 0.02 (0.01–0.07); *p* < 0.001), and thallium (0.42 (0.25–0.64) vs. 0.27 (0.18–0.39); *p* = 0.007). The levels of Gold were lower in the ASD group compared to the control group (0 (0–0.01) vs. 0.01 (0.01–0.02); *p* < 0.001). Additionally, differences were found in the titanium, arsenic, antimony, and cesium levels; however, the differences were not significant after correction. The medians, with interquartile range (IQR) range, and *p*-values for other trace elements are presented in Table 1 and Table 2.

#### 2.2.2. Comparison of Metal Concentrations between Children with HF-ASD and Healthy Controls

When comparing the levels of six trace metals (out of 30) in urine per creatinine between the group with HF-ASD and the control group, statistically significant differences were observed. The levels of two trace elements were higher in the HF-ASD group compared to the control group: rubidium (2265.4 (1655.7–2685.6) vs. 1395.1 (752.9–2113.3); *p* = 0.010) and silver (0.09 (0.07–0.14) vs. 0.02 (0.01–0.07); *p* < 0.001). The levels of four trace elements were lower in the HF-ASD group compared to the control group: titanium (15.53 (9.09–22.26) vs. 28.09 (18.84–39.00); *p* < 0.001), copper (7.54 (5.66–9.22) vs. 11.59 (8.66–15.60); *p* = 0.005), gold (0 (0–0.01) vs. 0.01 (0.01–0.02); *p* < 0.001), and lead (0.52 (0.32–0.75) vs. 0.83 (0.55–1.42); *p* = 0.03). Additionally, differences were found in the levels of beryllium, magnesium, aluminum, vanadium, chrome, manganese, nickel, and cadmium; however, the differences were not significant after correction. The medians, with IQR range, and *p*-values for other trace elements are presented in Table 1 and Table 2.

#### 2.2.3. Comparison of Metal Concentrations between Children with ASD and Children with HF-ASD

When we compared the levels of metals in the urine expressed per creatinine between children with HF-ASD and ASD, we found a significant difference in the level of one trace element. Specifically, the ASD group had higher levels of lead. This unique finding underscores the distinct metal profiles of these two groups.

#### 2.2.4. Comparison of Metal Concentrations between Children with ASD or HF-ASD Grouped by Deficit (Mild, Moderate, and Severe)

In our Comparison of metal levels in urine per creatinine between the groups with HF-ASD with mild, moderate, and severe deficits, we found some significant differences. Notably, when comparing the mild and moderate groups, the mild group had higher levels of calcium (*p* = 0.046) and strontium (*p* = 0.041), while the moderate group had higher zinc levels (*p* = 0.018).

When comparing the mild and severe deficit groups, we found higher levels of gallium (*p* = 0.022) and cadmium (*p* = 0.048) in the mild deficit group and higher levels of beryllium (*p* = 0.0044) in the severe deficit group.

When comparing the moderate and severe deficit groups, we found higher levels of gallium (*p* = 0.022) in the severe deficit group. However, after Bonferroni correction, none of the results were statistically significant.

#### 2.2.5. Comparison of Metal Concentrations between Children with ASD or HF-ASD with Epilepsy and without Epilepsy

When comparing the levels of metals in urine per creatinine between the first group of children with ASD or HF-ASD without epilepsy and the second group of children with ASD or HF-ASD with epilepsy, we found three trace elements that were statistically significantly higher in children with ASD or HF-ASD with epilepsy: manganese (*p* = 0.019), gold (*p* = 0.004), and lead (*p* = 0.05). However, it is important to note that none of these differences were statistically significant after Bonferroni correction.

### 2.3. Correlations between Metal Concentrations in Urine with Brain MRI Findings

All MRI indexes of ventricular volume, except the Evens and Bicaudate Frontal, had statistically significant correlations between them (Table 3). The Schiermans index, as expected, had a negative correlation with the other MRI indexes based on the calculation method. Cesium and beryllium were correlated with all seven MRI indexes. The trace elements chrome, cobalt, copper, rubidium, thallium, and lead were correlated with six MRI indexes. Vanadium, silver, and gold were correlated with five indices. Selenium was correlated with four MRI indexes. Cadmium and mercury were correlated with three indexes. Calcium and titanium were correlated with two indexes. The other trace elements were not correlated with MRI indexes. The highest correlations were seen between the bicaudate temporal index and chromium (0.704; *p* < 0.001), bicaudate index and chromium and (0.674; *p* < 0.001), and the bicaudate temporal index and cesium (0.664; *p* < 0.001). We compared the values of trace elements to the 95th percentile calculated from the control groups. A total of 21 out of 28 children with ASD or Asperger’s had at least 1 elevated trace element out of the 30; the maximum number of elevated trace elements was found in 1 child who had levels of 22 out of the 30 trace elements above the 95th percentile. When comparing the number of elevated trace elements to the MRI indexes, seven indexes were positively correlated (five of them strongly). The highest correlation was found in the bifrontal index 0.574; *p* = 0.001). The correlations remained even after essential elements were excluded.

Magnesium (−0.425, *p* = 0.029) levels were negatively correlated with the autism severity score (mild = 1, moderate = 2, severe = 3). The Huckman (0.410; *p* = 0.030) and Evans (0.397; *p* = 0.036) indexes were positively correlated with the presence of epilepsy in children with ASD or HF-ASD. Other MRI indexes were not statistically significant.

## 3. Discussion

### 3.1. Essential Metals

Calcium is one of the essential nutritional elements that is required for many vital physiological functions [11]. Impaired calcium in astrocytes (astrocyte-specific IP3R2 deficiency) was associated with ASD-like behavior in mice (social interaction defects and repetitive behaviors) [12]. Our study found no differences in the excreted levels of calcium in urine in the children with ASD, HF-ASD, and the control group. Additionally, no strong correlations were found between the ventricular volume indexes. However, the calcium concentration in urine cannot be used to estimate the differences in intracellular calcium. The second most abundant intracellular metal is magnesium. Elevated magnesium levels during pregnancy (in the fourth quartile) were associated with ADHD in children [13]. However, in a meta-analysis performed by Saghazadeh et al. [6], measuring magnesium in the hair of patients with ASD showed decreased magnesium values compared with that of controls (*p* = 0.007) [6]. Additionally, in another study, 17.6% of children with ASD were found to have magnesium deficiency, while calcium deficiency was observed in only 5.8% of cases. In the same study, deficits of other essential metals were equal to or smaller than 2%. Calcium and magnesium deficiencies are age-dependent [14]. Our study found no differences between the magnesium concentrations in urine when compared to the control group. However, we found a negative correlation between the autism deficit score and magnesium levels. Additionally, deficiencies of other trace elements, including zinc, manganese, molybdenum, and selenium, in the hair were associated with ASD [15,16]. Our study found no deficiencies in zinc, molybdenum, and selenium in children with ASD or HF-ASD compared to children in the control group. Manganese concentrations were higher in the ASD group compared with the HF-ASD and control groups. However, after Bonferroni correction, this was no longer statistically significant. Additionally, the correlations with ventricular volume indexes were low, although the correlations with selenium were statistically significant. Higher concentrations were associated with larger ventricular spaces.

Rahbar et al. [17] found a significant inverse correlation between molybdenum levels in hair and age (*p* < 0.001). They also found that lead, molybdenum, and manganese levels measured in hair were inversely correlated (*p* < 0.05) [17]. Exposure to trace metals can have an impact even during pregnancy. Schmid et al. [18] showed that antenatal use of iron supplements was associated with a lower risk of ASD development [18]. In our study, controls had higher Iron levels compared to children with ASD and HF-ASD; however, the differences were not significant.

In the study by Fang et al. [19], significant associations were found between ASD and copper and vanadium levels: children with ASD had lower VA and Cu levels and a lower Zn/Cu ratio than controls. Copper levels had a slight association with core ASD symptoms; for example, children with ASD had more GI comorbidities and sleep abnormalities. GI comorbidities and the levels of VA were also correlated [19]. Similarly, Macedoni et al. [20] found a significantly elevated Cu/Zn ratio in children with ASD compared to a control group of children with other neurological disorders [20]. In our study, copper levels were lowest in the HF-ASD group. The results were statistically significant compared to the ASD and control groups. Additionally, copper levels were lower in the ASD group than in the control group, although the results were not statistically significant. Vanadium levels were highest in the ASD group; the difference with the control group was statistically significant even after Bonferroni correction. A significant correlation was also seen between vanadium and brain MRI indexes. However, the same number of positive correlations was also seen in the correlations between ventricular volume indexes and copper.

### 3.2. Specific Trace Elements

Higher levels of titanium were found in healthy controls than in children in the ASD group (*p* = 0.18 after Bonferroni correction) and the HF-ASD group (*p* < 0.001). However, titanium levels did not correlate well with the ventricular volume indexes (only two of seven were correlated). In contrast, a study in Tunisia showed higher titanium levels in children with ASD [21].

We found that antimony levels were highest in the ASD group, although this was not statistically significant after Bonferroni correction. This concurs with another study in which antimony was positively correlated with autism behavior checklist scores [22]. The lack of correlation between structural brain changes (the volumes of ventricles) and antimony levels raises interesting questions about the causation between antimony and behavior checklist scores, which could have significant implications for our understanding of ASD.

The potential indirect effects of certain metals on the child during pregnancy, such as maternal thallium exposure at delivery, have significant implications for our understanding of ASD. This exposure has been found to have a negative impact on cord serum vitamin D levels, and vitamin D deficiency has been associated with an increased risk of cognitive impairment and behavior characteristic of ASD [19]. Our finding of higher levels of thallium in children with ASD compared to controls, which was statistically significant, and the ventricular volume indexes correlated in six cases with thallium levels, further underscores the potential impact of our research on the field.

Abd Wahli et al. [23] found lower urinary concentrations of lead in children with ASD compared with normally developing children. For this, the authors proposed that children with ASD might have a decreased ability to excrete heavy metals (including Pb), and as a consequence, toxic metals accumulate in the body and cause harm to the brain structure [23]. Results from animal models of developmental lead exposure showed some promising results of chelation therapy that could alleviate certain types of lead-induced cognitive dysfunction in ADHD [24]. However, lead levels after diagnosis are affected by many factors, including dietary factors (eating watermelons, lamb, and cold breakfasts such as cereals) [25]. Our study found the highest lead levels in children with ASD and the lowest in children with HF-ASD; the difference between the HF-ASD and the control groups was statistically significant. Rahbar et al. [17] found lower values of lead in the blood of children with ASD; however, after excluding cofounder effects, this difference was not statistically significant. Lead was the only metal that had a marginally significant lower blood concentration in ASD cases compared to TD controls (*p* = 0.05). However, after adjusting for potential confounding variables such as maternal age, father’s education level, and socioeconomic status, this difference was not statistically significant at a 5% significance level (*p* = 0.16) [17]. In a meta-analysis, the ASD group had higher concentrations of cadmium [26]; however, in our study, there was no difference in cadmium levels between the ASD group and the control group. In contrast, the lowest values were seen in the HF-ASD group.

In hair, a correlation was found between increasing ASD severity and increasing mercury concentrations in the hair among study participants diagnosed with moderate-to-severe ASD. A mechanical cause could be that MeHg can readily pass through the placenta and the blood–brain barrier (BBB) and cause damage to brain tissue. Therefore, it is more neurotoxic than inorganic mercury, which moves through membranes more [27]. In a study in Spain, however, mercury neurotoxicity was not shown to be associated with ASD [28]. Our study also found the highest mercury levels in the ASD group, but this was not statistically significant and correlated with only three ventricular volume indexes.

### 3.3. General Discussion

Different studies have found different results when comparing different trace elements; A meta-analysis by Dickerson et al. found conflicting associations between ASD and metal exposure, including 11 on aluminum, six on antimony, 15 on arsenic, five on beryllium, 17 on cadmium, 11 on chromium, 25 on lead, 14 on manganese, and 13 on nickel with markers of exposure in hair, urine, blood, teeth, fingernails, and air pollution. Only studies on cadmium and lead yielded the highest proportions of positive results (72% and 36%, respectively) [29]. However, healthy controls had the highest cadmium levels in our study. Another cause of the discrepancies between the results could be sex differences, which were described in another study [29].

In ASD, some structural changes are seen in the brain; for example, ASD is accompanied by significant between-group differences in cortical thickness (e.g., in frontotemporal and cingulate regions), and the individual’s total degree of neuroanatomical abnormality was significantly correlated with measures of symptom severity, as well as with the polygenic risk for ASD and other psychiatric conditions [30]. In our study, we could not prove this correlation between autism deficit scores and MRI indexes but found a correlation between two linear MRI indexes and the presence of epilepsy in children with ASD. Further, a computer-aided diagnosis system that uses features from structural MRI (sMRI) and resting state functional MRI (fMRI) to help predict an autism diagnosis is being developed [31]. Additionally, intraventricular spaces and hydrocephalus were associated with ASD. For example, a strong co-occurrence of ASD and hydrocephalus was found in a large population [32]. Furthermore, grey matter reductions (temporal, frontal lobes and abnormalities in the hippocampal and amygdala regions were observed in ASD [33].

Postema et al. [34] found that ASD was also significantly associated with alterations in cortical thickness asymmetry mainly in the medial frontal, orbitofrontal, cingulate, and inferior temporal areas and with asymmetry of the orbitofrontal surface area. These differences generally involved reduced asymmetry in individuals with ASD compared to controls. Furthermore, putamen volume asymmetry was significantly increased in ASD [34].

## 4. Materials and Methods

### 4.1. Study Cohort

Children in the study group were diagnosed with ASD or HF-ASD by a multi-disciplinary team consisting of expert pediatricians, neuropsychiatrists, and psychologists using a clinical assessment and a psychological assessment based on the criteria provided by Diagnostic and statistical manual of mental disorders (DSM-5). The children diagnosed with ASD were additionally grouped into three subgroups—1: mild deficit (1A, 2A); 2: moderate deficit (1B, 2B); and 3: severe deficit (2C, 3C)—that are based on the classification of behavior proposed by the Slovene educational authority, and covers impairments of social communication and interaction (assigned 1 for mild, 2 for moderate, and 3 for severe) on the one hand and the presence of impairment in behavioral flexibility (assigned A for mild, B for moderate, and C for severe) on the other hand [35,36].

Inclusion criteria: In the first part of the study, children with ASD or HF-ASD treated at the tertiary outpatient clinic were included if written permission was obtained from their parents. Children with disease-causing genetic mutations were excluded. A group of children and adolescents without any neurological problems or any other chronic disease was included in the control group after permission was obtained. For the second part of the study, children from the previous group who had clinical indications for brain MRI imaging were included.

The study protocol was approved by the National Medical Ethics Committee (0120-201/2016-2 KME 78/03/16).

### 4.2. Laboratory Evaluation of Urine

We obtained a second-morning urine specimen from all participants. Urine samples were aliquoted immediately after collection into two tubes and frozen at a temperature of −80 °C pending analysis. One tube was used for trace element analysis and the other for creatinine determination.

**Figure 2:**
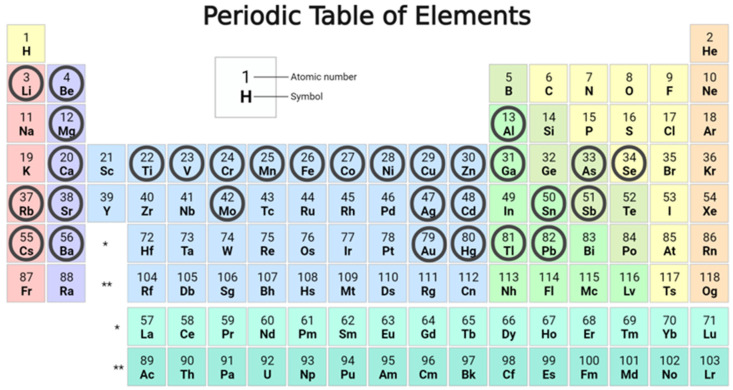
Periodic table of elements. Circles mark the trace elements that were measured in urine in our study. Legend: * Lanthanides, ** Actinides. The figure was created using BioRender.com (BioRender, Toronto, ON, Canada).

The trace elements Mn, Co, Cu, Zn, Se, Mo, Li, Be, V, Cr, Ni, Ga, As, Rb, Sr, Ag, Cd, Sn, Cs, Au, Hg, Tl, Pb, and U were measured (Figure 2) at the Institute of Clinical Chemistry and Biochemistry at the University Medical Centre Ljubljana. Measurements of prepared samples, calibrators, and control samples were taken on an Octapole Reaction System Inductively Coupled Plasma Mass Spectrometer (7700x, Agilent Technologies, Santa Clara, CA, USA). Separately, 0.2 mL aliquots of urine, calibrator, and control samples were mixed with 2 mL of ammonium hydroxide solution (Fluka Analytical TraceSELECT Ultra, Merck KGaA (Merck Group), Darmstadt, Germany) containing Triton X-100 (Aldrich Chemistry, Trace Metal Basis, Darmstadt, Germany), 1-butanol (Sigma Aldrich, ACS reagent, Darmstadt, Germany), ethylenediaminetetraacetic acid disodium salt dehydrate (Aldrich Chemistry, Trace Metal Basis), and an internal standard solution containing Bi, Ge, In, Li6, Lu, Rh, Sc, and Tb (Agilent Technologies, ICP-MS Internal Std Mix).

Limits of blank (LoB), limits of detection (LoD), and limits of quantification (LoQ) of all trace elements were based on Clinical and Laboratory Standards Institute (CLSI) recommendations with a classical approach using the reference material Seronorm Trace Elements Urine L-1 and L-2 (Ref. no: 201605 and 201705, Sero) in different dilutions to obtain minimally positive samples [37].

Urinary creatinine was measured using the Roche reagent kit on a Roche Modular P analyzer (Roche Diagnostics GmbH, Mannheim, Germany). All tests were performed following the instructions of the kit provider.

### 4.3. Calculation of Linear Indexes of the Intracranial Fluid Spaces

For the second part of the study, we retrospectively collected anonymized dicom files of brain MRI studies performed routinely on the patients included in the first part of the study. From the first group, 21 children with ASD and five children with HF-ASD had available brain MRI dicom files. None of the healthy controls had routine brain MRIs performed because they had no health issues needing MRI brain imaging. The dicom files were analyzed using the program 3D slicer [38]. For the evaluation of the intracranial ventricular system, we measured and calculated seven linear indexes, as reported by Wilk et al. [39]:

- Evans’ Index (maximum distance between anterior horns divided by maximum internal skull diameter),
- Bifrontal Index (the maximum distance between anterior horns divided by the maximum internal diameter of the frontal bone),
- Bicaudate Index (Minimum bicaudate nuclei distance divided by internal skull diameter measured along the same line),
- Bicaudate-Frontal Index (the minimum bicaudate nuclei distance divided by the maximum distance between anterior horns),
- Bicaudate-Temporal Index (the minimum bicaudate nuclei distance divided by maximum internal skull diameter),
- Schiersmann’s Index (the maximum external diameter of the skull divided by cella media distance),
- Huckman Number (the sum of the maximum distance between anterior horns and minimum bicaudate nuclei distance) [39].

### 4.4. Data Analysis

Data were collected in Microsoft Excel (Microsoft Corporation. (2018). Microsoft Excel. Redmond, Washington, USA. Retrieved from https://office.microsoft.com/excel, accessed on 17 July 2024). All statistical analyses were performed using SPSS 22.0 (IBM SPSS Statistics for Windows, Version 22.0. Armonk, NY: IBM Corp., USA). The graphs were drawn with SPSS 22.0. Differences between the groups were determined statistically using a nonparametric test (Mann–Whitney test, two-tailed) followed by Bonferroni correction. *p*-values < 0.05 were taken as statistically significant. Correlations were calculated using Pearson’s correlation (two-tailed), and *p* < 0.05 was taken as statistically significant. The average values were calculated based on the control group (C), whereas the 5th and 95th percentiles were calculated using weighted averages in IBM SPSS 22.0. The values from the two study groups were then directly compared (higher, lower) to the calculated 95th percentile.

## 5. Conclusions

Excessive exposure to toxic heavy metals after conception and during childhood, and inadequate intake of essential metal elements may be associated with ASD [4]. Our study found some differences in the concentrations of toxic elements in children with ASD compared with children in the control group. Most were higher in the ASD group, but some were higher in the control group (titanium and gold). Many studies follow different approaches and tissue analyses, yielding different results. Our study confirmed some results, but some are nonspecific or even different from those previously described. Additionally, cations can be further impacted by other elements, for example, chronic fluorine exposure and aluminum’s free metal action reinforce the pathological symptoms of ASD [40]. The relatively small sample of patients could have caused this. However, the main point was confirmed: exposure to toxic metals is present. The impact on MRI ventricular volume and brain atrophy was seen; however, as the MRI scans did not correspond to the date of the laboratory exams, the results need to be proven. Additionally, because they were healthy, the control group did not need an MRI exam; therefore, a negative control group is missing in confirming the importance of our findings. Laboratory examination of toxic metals was also performed after diagnosis; therefore, we cannot confirm that the cumulative effect of toxic exposure before ASD developed and the cumulative effect of habits present in children with ASD (e.g., eating specific foods [41]) were the cause of the differences. In the end, correcting poor dietary behaviors and preventing excessive exposure to toxic metals may be beneficial for preventing and treating the disease [4].

Further multicenter studies evaluating genetic, epidemiological, and structural changes in combination are needed.

## Figures and Tables

**Table 1:** Median values with the 25th and 75th percentiles of different trace elements (expressed per creatinine) in urine for the different study groups.

Element	Group A1 (*n* = 107)	Group B1 (*n* = 38)	Group C1 (*n* = 49)
Li	23.3 (14.4–50.8)	23.6 (13.9–30.7)	24.5 (17.4–30.9)
Be	0.02 (0.02–0.05)	0.02 (0.01–0.03)	0.03 (0.01–0.07)
Mg	86,531.7 (51,554.7–149,422.6)	81,625.5 (56,748.3–121,036.7)	136,982.1 (61,908.1–195,288.7)
Al	3.40 (1.57–8.61)	2.00 (1.05–5.31)	4.78 (1.82–9.29)
Ca	4860.7 (2130.3–10147.1)	5417.9 (2803.7–9516.8)	6616.5 (3585.8–10672.0)
Ti	17.65 (10.19–33.29)	15.52 (9.09–22.25)	28.09 (18.83–39.00)
V	0.09 (0.06–0.15)	0.06 (0.04–0.14)	0.05 (0.02–0.08)
Cr	0.25 (0.18–0.49)	0.18 (0.12–0.32)	0.33 (0.21–0.48)
Mn	0.43 (0.33–0.73)	0.29 (0.23–0.53)	0.44 (0.30–0.62)
Fe	2.77 (0.06–6.62)	2.34 (0.83–4.44)	3.80 (1.93–5.69)
Co	0.4 (0.2–1.0)	0.5 (0.3–0.7)	0.6 (0.3–1.0)
Ni	0.56 (0.29–1.60)	0.38 (0.28–0.96)	0.83 (0.36–1.51)
Cu	9.69 (6.49–18.25)	7.54 (5.66–9.22)	11.59 (8.65–15.59)
Zn	385.88 (249.26–639.53)	462.83 (289.25–629.64)	487.26 (292.32–699.29)
Ga	0.03 (0.02–0.06)	0.02 (0.02–0.04)	0.03 (0.02–0.06)
As	6.74 (3.43–15.60)	4.93 (2.46–15.95)	4.35 (2.64–9.10)
Se	23.91 (15.25–32.38)	20.09 (16.98–25.07)	23.2 (16.87–27.45)
Rb	2682.9 (1581.3–4750.4)	2265.4 (1655.7–2685.6)	1395.1 (752.9–2113.2)
Sr	103.72 (57.69–207.41)	103.71 (70.59–169.62)	130.66 (77.67–173.92)
Mo	71.88 (41.44–111.45)	60.12 (42.02–84.22)	65.68 (44.76–92.33)
Ag	0.12 (0.09–0.19)	0.09 (0.06–0.13)	0.02 (0.01–0.06)
Cd	0.09 (0.06–0.15)	0.07 (0.05–0.11)	0.11 (0.08–0.16)
Sn	0.38 (0.16–1.04)	0.27 (0.12–0.69)	0.34 (0.21–0.61)
Sb	0.06 (0.04–0.13)	0.05 (0.03–0.07)	0.05 (0.02–0.07)
Cs	8.39 (5.55–13.64)	7.54 (5.32–9.01)	7.09 (4.64–9.05)
Ba	2.07 (0.99–4.79)	2.82 (1.35–5.07)	2.75 (1.7–5.29)
Au	0 (0.00–0.01)	0 (0.00–0.01)	0.01 (0.01–0.02)
Hg	0.13 (0.05–0.28)	0.165 (0.08–0.38)	0.15 (0.07–0.28)
Tl	0.42 (0.25–0.64)	0.32 (0.23–0.38)	0.27 (0.18–0.39)
Pb	0.73 (0.52–1.31)	0.52 (0.32–0.74)	0.83 (0.54–1.42)
U	0.01 (0.00–0.01)	0.01 (0.00–0.01)	0.01 (0.00–0.01)

**Table 2:** The *p*-values from the comparison of the 3 study groups. In the brackets are values obtained after Bonferroni correction.

	Group A1 vs. Group C1	Group A1 vs. Group B1	Group B1 vs. Group C1
Li	0.750 (1.000)	0.400 (1.000)	0.584 (1.000)
Be	0.664 (1.000)	0.011 (0.33) *	0.020 (0.600) *
Mg	0.096 (1.000)	0.500 (1.000)	0.020 (0.600) *
Al	0.325 (1.000)	0.033 (0.99) *	0.011 (0.330) *
Ca	0.144 (1.000)	0.472 (1.000)	0.436 (1.000)
Ti	0.006 (0.180) *	0.244 (1.000)	<0.001 (<0.001) **
V	<0.001 (<0.001) **	0.037 (1.000) *	0.011 (0.330) *
Cr	0.357 (1.000)	0.003 (0.090) *	0.002 (0.060) *
Mn	0.387 (1.000)	0.002 (0.060) *	0.045 (1.000) *
Fe	0.194 (1.000)	0.927 (1.000)	0.094 (1.000)
Co	0.166 (1.000)	0.618 (1.000)	0.279 (1.000)
Ni	0.505 (1.000)	0.085 (1.000)	0.019 (0.570) *
Cu	0.397 (1.000)	0.006 (0.180) *	<0.001 (0.005) **
Zn	0.228 (1.000)	0.607 (1.000)	0.555 (1.000)
Ga	0.705 (1.000)	0.004 (0.120) *	0.023 (0.690) *
As	0.038 (1.000) *	0.551 (1.000)	0.360 (1.000)
Se	0.425 (1.000)	0.106 (1.000)	0.342 (1.000)
Rb	<0.001 (<0.001) **	0.069 (1.000)	<0.001 (0.010) **
Sr	0.805 (1.000)	0.679 (1.000)	0.426 (1.000)
Mo	0.713 (1.000)	0.251 (1.000)	0.342 (1.000)
Ag	<0.001 (<0.001) **	0.055 (1.000)	<0.001 (<0.001) **
Cd	0.156 (1.000)	0.098 (1.000)	0.010 (0.300) *
Sn	0.96 (1.000)	0.228 (1.000)	0.241 (1.000)
Sb	0.011 (0.330) *	0.112 (1.000)	0.561 (1.000)
Cs	0.010 (0.300) *	0.083 (1.000)	0.419 (1.000)
Ba	0.064 (1.000)	0.454 (1.000)	0.411 (1.000)
Au	<0.001 (0.001) **	0.040 (1.000) *	<0.001 (<0.001) **
Hg	0.267 (1.000)	0.230 (1.000)	0.821 (1.000)
Tl	<0.001 (0.007) **	0.010 (0.300) *	0.249 (1.000)
Pb	0.826 (1.000)	<0.001 (0.010) **	0.001 (0.03) **
U	0.648 (1.000)	0.672 (1.000)	0.623 (1.000)

Legend: * Statistically significant results before correction. ** Statistically significant results after Bonferroni correction.

**Table 3:** (**A**) The calculated Pearson correlation factors between the values in the rows and columns. (**B**) The calculated Pearson correlation factors between the values in the rows and columns.

(A)										
	Huckman Index	Evans Index	Bifrontal Index	Bicaudate Frontal Index	Bicaudate Index	Bicaudate Temporal Index	Schiersmann Index	Number of Statistically Significant Correlations	Autism Deficit Score	Epilepsy Present
Li	0.294	0.324	0.343	0.250	0.309	0.340	−0.087	0	0.228	0.094
Be	0.410 *	0.488 **	0.572 **	0.523 **	0.606 **	0.615 **	−0.395 *	7	0.114	0.210
Mg	0.216	0.209	0.369	0.078	0.120	0.158	−0.194	0	−0.425 *	−0.009
Al	0.060	0.288	0.307	0.200	0.217	0.268	0.114	0	0.218	−0.059
Ca	0.308	0.148	0.207	0.411 *	0.371	0.383 *	−0.180	2	−0.177	−0.023
Ti	0.194	0.382 *	0.494 **	0.198	0.263	0.302	−0.326	2	−0.189	−0.114
V	0.529 **	0.511 **	0.556 **	0.266	0.407 *	0.453 *	−0.311	5	−0.189	0.314
Cr	0.644 **	0.610 **	0.517 **	0.511 **	0.674 **	0.704 **	−0.251	6	0.000	0.515 *
Mn	0.238	0.287	0.455 *	0.160	0.228	0.259	−0.132	1	−0.169	0.154
Fe	0.022	0.049	0.284	0.001	−0.026	0.020	−0.053	0	−0.222	−0.045
Co	0.428 *	0.459 *	0.416 *	0.431 *	0.486 **	0.555 **	−0.347	6	−0.280	0.233
Ni	0.061	0.249	0.093	0.139	0.099	0.218	−0.034	0	−0.061	−0.105
Cu	0.374	0.488 **	0.482 **	0.408 *	0.503 **	0.530 **	−0.419 *	6	−0.168	0.110
Zn	0.246	0.149	0.063	0.269	0.225	0.265	−0.314	0	−0.206	−0.018
Ga	0.059	0.141	0.369	0.053	0.062	0.101	−0.063	0	−0.153	−0.009
As	−0.065	−0.210	−0.056	−0.129	−0.127	−0.175	0.252	0	−0.281	−0.128
Se	0.386 *	0.432 *	0.376 *	0.257	0.345	0.390 *	−0.325	4	−0.195	0.143
Rb	0.464 *	0.471 *	0.468 *	0.458 *	0.549 **	0.581 **	−0.355	6	−0.223	0.301
Sr	0.300	0.338	0.368	0.290	0.310	0.369	−0.313	0	−0.255	−0.082
Mo	0.189	0.269	0.242	0.111	0.164	0.218	−0.035	0	−0.136	0.237
Ag	0.530 **	0.471 *	0.526 **	0.303	0.422 *	0.466 *	−0.340	5	−0.178	0.336
Cd	0.159	0.285	0.311	0.429 *	0.436 *	0.467 *	−0.187	3	−0.006	0.137
Sn	0.090	0.030	−0.148	0.244	0.196	0.216	−0.201	0	−0.118	0.216
Sb	0.171	0.218	0.090	0.070	0.057	0.136	−0.271	0	0.037	−0.070
Cs	0.576 **	0.597 **	0.526 **	0.495 **	0.618 **	0.664 **	−0.403 *	7	−0.164	0.350
Ba	0.308	0.186	0.223	0.351	0.308	0.346	−0.229	0	−0.184	−0.135
Au	0.526 **	0.622 **	0.578 **	0.292	0.424 *	0.510 **	−0.318	5	−0.077	0.261
Hg	0.374 *	0.346	0.206	0.338	0.393 *	0.428 *	−0.359	3	0.161	0.209
Tl	0.374 *	0.390 *	0.402 *	0.380 *	0.427 *	0.478 *	−0.240	6	−0.309	0.167
Pb	0.402 *	0.406 *	0.548 **	0.393 *	0.476 *	0.499 **	−0.151	6	−0.041	0.260
U	0.121	0.190	0.339	0.045	0.032	0.111	−0.104	0	−0.126	−0.044
Starost_MR	0.123	0.013	−0.209	0.197	0.135	0.174	0.137	0	−0.196	0.104
Starost	0.019	−0.058	−0.320	0.143	0.081	0.110	0.197	0	−0.272	0.264
Huckman index	1	0.785 **	0.710 **	0.624 **	0.790 **	0.826 **	−0.597 **	7	−0.153	0.410 *
Evans index	0.785 **	1	0.803 **	0.327	0.600 **	0.683 **	−0.536 **	6	−0.052	0.397 *
Bifrontal index	0.710 **	0.803 **	1	0.408 *	0.650 **	0.649 **	−0.448 *	7	0.053	0.281
Bicaudate frontal indec	0.624 **	0.327	0.408 *	1	0.928 **	0.909 **	−0.422 *	6	0.059	0.188
Bicaudate index	0.790 **	0.600 **	0.650 **	0.928 **	1	0.979 **	−0.517 **	7	0.065	0.364
Bicaudate temporal index	0.826 **	0.683 **	0.649 **	0.909 **	0.979 **	1	−0.537 **	7	0.017	0.357
Schiersmann index	−0.597 **	−0.536 **	−0.448 *	−0.422 *	−0.517 **	−0.537 **	1	7	0.150	−0.107
**(B)**										
	**Huckman Index**	**Evans Index**	**Bifrontal Index**	**Bicaudate Frontal Indec**	**Bicaudate Index**	**Bicaudate Temporal Index**	**Schiersmann Index**	**Number of Statistically Significant Correlations**	**Autism Deficit Score**	**Epilepsy Present**
Number of elevated metal concentrations	0.515 **	0.535 **	0.574 **	0.389 *	0.498 **	0.544 **	−0.429 *	7 (5 of them strong (*p* < 0.01, two-tailed)	−0.222	0.200
Number of elevated metals, excluding essential	0.532 **	0.549 **	0.587 **	0.370 *	0.488 **	0.534 **	−0.412 *	6 (5 of them strong (*p* < 0.01, two-tailed)	−0.202	0.200

Legend: * Statistically significant results (*p* < 0.05). ** *p* < 0.01.

## Data Availability

The data presented in this study are available on request from the corresponding authors.

## Supplementary Materials

The information can be downloaded at: https://www.mdpi.com/article/10.3390/ijms25158050/s1.
