# Supplementary material for: Association between Autism Spectrum Disorder, Trace Elements, and Intracranial Fluid Spaces
Source: Int J Mol Sci. 2024 Jul 24;25(15):8050. doi: 10.3390/ijms25158050 (PMC11311321; doi:10.3390/ijms25158050)
Supplement: Supplementary file 1 [file ijms-25-08050-s001.zip › ijms-3069960-supplementary.pdf]

Supplementary file - **Toxicological profiles of trace elements**

Element	Sources of exposure		Health effects	Regulatory limits	Additional
	Occupational	Environmental			
Li		Exposure to lithium in the environment, such as through drinking water or food, is typically at very low levels and is not associated with adverse health effects.	Exposure to lithium is generally considered to be safe and can even have positive effects on mental health when used as a medication for certain conditions. Lithium is a naturally occurring element and is used primarily as a medication to treat bipolar disorder and certain mood disorders. While therapeutic use of lithium is generally safe, there can be side effects associated with its use, including gastrointestinal symptoms, hand tremors, and thyroid issues.		Lithium exposure through prescribed medication under proper medical supervision is generally safe and can provide significant benefits for individuals with mood disorders. However, misuse or overdose can have serious health consequences.
Be	Occupational exposure to beryllium occurs primarily in industries where beryllium and its compounds are used. This includes industries involved in beryllium mining, extraction, machining, electronics, aerospace, nuclear power, and defense.		Chronic Beryllium Disease (CBD): This is the most well-known and serious health effect associated with beryllium exposure. CBD is a chronic lung disease caused by inhaling beryllium particles or fumes. It is characterized by lung inflammation, cough, chest pain, and breathing difficulties. CBD is often progressive and may result in permanent lung damage. Acute Beryllium Disease: Some individuals may experience an acute form of beryllium disease following high levels of exposure. Symptoms include fever, cough, and difficulty breathing. Acute beryllium disease can progress to CBD.	Workers in industries with potential beryllium exposure should follow safety protocols and use personal protective equipment to minimize exposure. Employers are required to adhere to safety regulations set by government agencies, such as OSHA (Occupational Safety and Health Administration) in the United States. Monitoring: Regular monitoring and testing	

			Skin Sensitization: Some individuals can develop an allergic skin reaction when their skin comes into direct contact with beryllium or beryllium-containing materials.<br>Cancer: Beryllium has been classified as a possible human carcinogen by the International Agency for Research on Cancer (IARC). While the link between beryllium exposure and cancer is not as strong as for other substances like asbestos, it is still a concern.	are important for individuals who are exposed to beryllium as part of their job. Early detection and intervention can help manage the health effects.	
Al	Workers in aluminum production, welding, and mining, may be at risk of occupational exposure to high levels of aluminum dust, fumes, or compounds.	Aluminum is a natural element present in the Earth's crust and can be found in soil, water, and air. The primary source of aluminum exposure for the general population is through food and water. Small amounts of aluminum naturally occur in many foods and can leach into water from aluminum cookware or packaging.	Ingested Aluminum: In most cases, dietary exposure to aluminum is not a cause for concern, as the body can efficiently eliminate small amounts. However, excessive consumption of aluminum-containing products, particularly antacids with high aluminum content, can lead to health issues, such as aluminum toxicity, which may result in bone and kidney problems.<br>Respiratory Effects: Occupational exposure to aluminum dust or fumes can lead to respiratory issues, including coughing and breathing difficulties. Prolonged exposure may contribute to lung problems, such as pulmonary fibrosis.<br>Neurological Effects: There has been some concern regarding the potential link between aluminum exposure and neurological disorders like Alzheimer's disease.	For dietary exposure, a balanced diet and avoidance of excessive use of aluminum-containing antacids or other products can help prevent overexposure.<br>In occupational settings, following safety guidelines and using personal protective equipment can help minimize aluminum exposure.<br>Overall, aluminum exposure is generally considered safe in the normal course of daily life.	

		Aluminum is also used in antacids, baking powder, and certain food additives.			
Ti	Workers in industries that involve titanium mining, refining, or manufacturing may be at risk of occupational exposure to titanium dust or fumes. Appropriate workplace safety measures are important to minimize exposure	Titanium is a naturally occurring element and is present in various rocks, soils, and sediments. It can be released into the environment through weathering and erosion.<br>Titanium dioxide (TiO_2) is one of the most common forms of titanium and is used in products like sunscreen, paint, and food colorings. It is generally considered safe for these applications.	Low Toxicity: Titanium is generally considered to have low toxicity. Acute exposure to titanium at normal environmental levels is not typically associated with severe health effects.<br>Biocompatibility: Titanium is known for its biocompatibility, making it a valuable material for medical devices such as implants (e.g., dental implants, joint replacements) that can be safely used within the human body.<br>Allergenic Potential: In some cases, individuals may experience allergic reactions to titanium-based materials, although this is relatively rare.	Regulatory agencies have established permissible exposure limits for titanium in the workplace to protect workers from excessive exposure.	Exposure to titanium is generally considered to be of low concern for health effects when encountered at typical environmental levels or through dietary intake. Occupational exposure to high levels of titanium should be managed through safety protocols to minimize potential risks.
V	Workers in industries such as mining, smelting, and the production of steel and	Vanadium is naturally present in some foods, and dietary intake can contribute to	Vanadium exposure is associated with potential health effects, including respiratory and gastrointestinal issues when inhaled or ingested in large quantities.		In areas with high natural levels of vanadium in the soil and water, there may be concerns about

	aluminum are at risk of exposure to vanadium through inhalation of vanadium-containing dust or fumes.	vanadium levels in the body. However, the amount of vanadium obtained from a typical diet is usually low and not a significant cause for concern.			vanadium contamination in drinking water.
Cr	Workers in industries such as chrome plating, stainless steel production, and leather tanning may be at risk of exposure to hexavalent chromium through inhalation of dust or fumes.	Hexavalent chromium can contaminate soil and water due to industrial activities, posing risks to local ecosystems and, potentially, human health when exposure occurs through drinking water or consumption of contaminated crops.	Respiratory Effects: Inhalation of hexavalent chromium can lead to respiratory problems, including coughing, wheezing, and lung cancer. Long-term occupational exposure is associated with a higher risk of lung cancer.<br>Skin Effects: Skin contact with hexavalent chromium can result in dermatitis, skin ulcers, and allergic reactions. Certain industrial workers are more susceptible to these skin effects.<br>Gastrointestinal Effects: Ingesting high levels of hexavalent chromium can lead to gastrointestinal distress.	Occupational Safety: Workers in industries with potential hexavalent chromium exposure should follow safety protocols and use personal protective equipment to minimize exposure. Employers are required to adhere to safety regulations set by government agencies to protect workers.<br>Regulatory Limits: Many countries have established strict regulatory limits on permissible levels of hexavalent chromium exposure in the workplace and the environment.<br>Dietary Chromium: Dietary intake of	Chromium III (Trivalent Chromium): This form of chromium is found in small amounts in food and is an essential nutrient for humans. It plays a role in glucose metabolism. Trivalent chromium is generally considered safe and not associated with significant health risks.<br>Chromium VI (Hexavalent Chromium): Hexavalent chromium is a more toxic form of chromium, and exposure to high levels can lead to adverse health effects. It is a known human carcinogen when inhaled.

				trivalent chromium is generally considered safe and is essential for health. However, excessive intake of chromium supplements may lead to adverse effects.	
Mn	Workers in certain industries, such as mining, welding, and the production of steel, batteries, and dry cell batteries, may be at risk of occupational exposure to high levels of manganese, particularly through inhalation of manganese dust or fumes.	Manganese is found in many foods, including nuts, grains, leafy vegetables, and tea. Dietary intake of manganese is generally not a health concern and is essential for normal growth and development.<br>Low levels of manganese are present in drinking water, and exposure through drinking water is generally not a significant source of exposure.	Respiratory Effects: Inhalation of manganese particles or fumes, especially in occupational settings, can lead to respiratory issues, such as coughing, wheezing, and shortness of breath.<br>Neurological Effects: Chronic exposure to high levels of manganese, particularly in the workplace, can lead to neurological effects. This condition is often referred to as manganism and has symptoms similar to Parkinson's disease, including tremors, muscle stiffness, and balance problems.<br>Cognitive and Behavioral Effects: Long-term exposure to high levels of manganese may be associated with cognitive and behavioral changes, particularly in occupational settings. These changes can include memory issues, mood swings, and irritability.<br>Developmental Effects: High levels of manganese exposure during pregnancy, such as from occupational settings, may potentially affect fetal development. Studies have suggested a possible link	Occupational Safety: Workers in industries with potential manganese exposure should follow safety protocols and use personal protective equipment to minimize exposure. Employers are required to adhere to safety regulations to protect workers.<br>Regulatory Limits: Many countries have established regulatory limits on permissible levels of manganese exposure in the workplace and the environment to protect workers and the general population.	

			between prenatal manganese exposure and developmental delays in children.		
Co	Workers in industries that involve the mining, refining, and production of cobalt and cobalt-containing materials, such as alloys, may be at risk of occupational exposure through inhalation of cobalt dust or fumes.	Cobalt-chromium alloys are commonly used in orthopedic implants and prosthetic devices, such as hip replacements. Long-term exposure to cobalt from these implants, due to wear and corrosion, can lead to localized health effects, including metallosis (a reaction to metal debris) and implant failure.	Exposure to cobalt can have various health effects, primarily in occupational settings with potential cobalt exposure through inhalation or skin contact. Lung Effects: Inhalation of cobalt dust or fumes can lead to respiratory issues, including "hard metal lung disease" or cobalt pneumoconiosis, which is a type of interstitial lung disease.<br>Skin Effects: Skin contact with cobalt-containing materials can cause contact dermatitis or skin allergies.<br>Cardiovascular Effects: Ingestion of high levels of cobalt, typically through contaminated food or drink, can result in cardiovascular issues, including cardiomyopathy (a disease of the heart muscle).<br>Allergic Reactions: Some individuals may experience allergic reactions to cobalt, which can manifest as skin rashes and respiratory symptoms.	Monitoring and controlling occupational exposure to cobalt are essential to protect the health of workers, and individuals with cobalt-containing implants should be monitored for potential health effects.	Exposure to cobalt, a metallic element, can have various health effects depending on the level and duration of exposure. Cobalt is an essential trace element required in small amounts for certain physiological processes, but exposure to high levels of cobalt, particularly cobalt dust or fumes in occupational settings, can lead to health concerns.
Ni	Workers in industries such as stainless steel production, nickel refining, welding, and electroplating may be at risk of occupational exposure to nickel	Nickel is found in some foods. Ingestion of dietary nickel is generally not a significant source of exposure, and most people do not experience	Allergic Contact Dermatitis: One of the most common health effects of nickel exposure is allergic contact dermatitis. This condition is characterized by red, itchy, and inflamed skin where nickel comes into contact with the body. It can occur in response to wearing nickel-containing jewelry, using nickel-plated items, or occupational exposure.	Occupational Safety: Workers in industries with potential nickel exposure should follow safety protocols and use personal protective equipment to minimize exposure. Employers are required to adhere to	Exposure to nickel can have both acute and chronic health effects, with skin allergies and respiratory issues being common. Prolonged occupational exposure to certain forms of nickel is associated with an

	through inhalation of nickel dust, fumes, or skin contact.	adverse effects from dietary nickel.	Respiratory Effects: Inhalation of nickel particles or fumes, particularly in occupational settings, can lead to respiratory problems, including coughing, shortness of breath, and chronic bronchitis.<br>Cancer: Prolonged occupational exposure to certain forms of nickel, such as nickel compounds like nickel subsulfide and nickel oxide, has been associated with an increased risk of lung cancer. Nickel compounds are classified as Group 1 human carcinogens by the International Agency for Research on Cancer (IARC).	safety regulations to protect workers.<br>Regulatory Limits: Many countries have established regulatory limits on permissible levels of nickel exposure in the workplace and the environment to protect workers and the general population.<br>Hypoallergenic Products: Some individuals with nickel allergies use hypoallergenic or nickel-free products to avoid skin contact with nickel.	increased risk of lung cancer. Preventive measures, safety protocols, and regulatory limits are crucial to reduce exposure to harmful levels of nickel. Individuals with concerns about nickel exposure or known nickel allergies should take precautions to avoid skin contact with nickel-containing items.
Cu	Certain occupational groups, such as copper miners, smelters, and workers in the copper manufacturing industry, may be at risk of exposure to elevated levels of copper through inhalation of copper dust or fumes.	Copper can be found in the environment due to industrial emissions and activities. Elevated copper levels in the environment can impact aquatic ecosystems and may pose a risk to aquatic life.<br>In homes with copper plumbing systems, there can be a potential for	High copper levels in drinking water can lead to gastrointestinal issues, such as stomach cramps, nausea, and diarrhea, particularly in individuals with copper sensitivities.<br>: Copper is one of the components of dental amalgam (silver fillings). In some cases, individuals may experience allergic reactions or sensitivity to the metals in dental amalgams.		For most people, dietary copper intake is not a concern, and copper is essential for maintaining good health

		copper to leach into drinking water, especially in areas with acidic water or old plumbing systems.			
Zn	Workers in certain industries, such as galvanizing, metal production, and battery manufacturing, may be at risk of occupational exposure to zinc. Appropriate workplace safety measures should be followed to minimize exposure.	Zinc is an essential nutrient, and a small amount of dietary zinc is required for normal growth and development. It is found in many foods, including meats, dairy products, nuts, and whole grains. Dietary intake of zinc is generally not a health concern, and it is important for maintaining overall health and immune function.	Immune Function: Zinc is necessary for a well-functioning immune system. Adequate zinc levels support the body's ability to combat infections and illnesses.<br>Growth and Development: Zinc is important for growth, particularly in infants and children. It is required for normal development, including wound healing and sexual maturation.<br>Cognitive Function: Adequate zinc intake supports cognitive function and can help with memory and attention.<br>Skin Health: Zinc is used in some skin care products to treat conditions like acne, as it has anti-inflammatory properties and can help regulate oil production in the skin.<br>Dietary Zinc Deficiency:<br>Inadequate dietary intake of zinc can lead to health issues. Zinc deficiency can result in growth retardation, delayed sexual maturation, weakened immune function, and skin problems.<br>Supplements and Overexposure:<br>While zinc is essential in small amounts, excessive intake of zinc through supplements or medications can lead to health issues. High-dose zinc supplements	Regulatory agencies have established limits on permissible levels of zinc exposure in the workplace to protect workers from excessive exposure.	Exposure to zinc is generally safe and essential for human health. Adequate dietary intake of zinc is important for overall well-being, immune function, and normal growth and development. While excessive zinc intake through supplements can have adverse effects, it is generally not a concern when obtained from a balanced diet. Occupational exposure to high levels of zinc should be managed through safety protocols to minimize health risks.

			can interfere with the absorption of other minerals and cause gastrointestinal problems.		
Ga	Workers in industries related to electronics, semiconductor manufacturing, and the production of certain medical devices may be at risk of occupational exposure to gallium. Exposure can occur through inhalation of gallium fumes or dust or skin contact.	Environmental exposure to gallium is generally not a significant concern for the general population, as gallium is not commonly found in the environment at levels that pose health risks.	Low Toxicity: Gallium is generally considered to have low toxicity. Acute exposure to gallium, even at relatively high levels, is not typically associated with severe health effects.<br>Skin Staining: Gallium can cause temporary skin staining or discoloration, especially when it comes into contact with the skin for extended periods. This is generally not harmful and can be easily removed.<br>Inhalation Risks: Inhalation of gallium fumes, particularly in industrial settings with high concentrations, may lead to respiratory irritation and potentially lung effects. However, such exposures are rare and typically associated with mishandling or improper workplace safety measures.	Regulatory agencies have established limits on permissible levels of gallium exposure in the workplace to protect workers from excessive exposure.	Exposure to gallium is generally considered to be of low concern for health effects, particularly at normal environmental exposure levels. Occupational exposure to gallium, while rare, should be managed through proper workplace safety protocols to minimize potential risks. Acute exposure to gallium is not typically associated with severe health effects, but safety measures should be followed to protect workers in relevant industries.
As	Some industrial settings, such as mining, smelting, and pesticide manufacturing, can expose workers to high levels of arsenic.<br>Occupational	Arsenic can naturally occur in soil and groundwater, and high levels of arsenic in drinking water and crops can pose risks to people living in	Acute Poisoning: High-level acute exposure to arsenic can result in symptoms such as severe abdominal pain, vomiting, diarrhea, and in severe cases, it can lead to coma or death.<br>Chronic Exposure: Long-term, low-level exposure to arsenic can have a range of chronic health effects, including:	Regulatory agencies have established limits on permissible levels of arsenic in drinking water and food to protect the public from excessive exposure.<br>In occupational settings with potential arsenic	Exposure to arsenic can have severe health effects, both acutely and chronically. Prevention measures include monitoring and treating arsenic-contaminated drinking water sources, regulating permissible

	exposure to arsenic can occur through inhalation of dust or fumes or skin contact.	areas with arsenic-contaminated water sources. Certain geographic regions have naturally high levels of arsenic in their water supply. Arsenic can be found in certain foods, especially rice and some seafood, due to its presence in the environment.	Cancer: Arsenic is classified as a Group 1 human carcinogen by the International Agency for Research on Cancer (IARC). Chronic exposure to arsenic in drinking water is associated with an increased risk of skin, bladder, and lung cancers.<br>Cardiovascular Effects: Arsenic exposure has been linked to cardiovascular issues, including heart disease and hypertension.<br>Skin Problems: Chronic exposure can lead to skin conditions, such as hyperpigmentation and the development of skin lesions.<br>Neurological Effects: Arsenic exposure may affect the nervous system, leading to neurological symptoms, including numbness, tingling, and memory problems.	exposure, safety protocols and personal protective equipment should be used to minimize risk.	levels of arsenic in food and water, and implementing safety measures in industries with potential arsenic exposure.
Se		Environmental exposure to selenium is generally not a significant concern for the general population. Elevated levels of selenium in drinking water or soil are more likely to affect local ecosystems and wildlife. Selenium is obtained	Selenium deficiency is rare in well-nourished populations but can occur in regions with selenium-poor soil or in individuals with specific medical conditions that hinder selenium absorption.<br>Selenium deficiency can lead to health problems, including muscle weakness, fatigue, impaired immune function, and a higher risk of certain diseases, such as Keshan disease (a form of cardiomyopathy) and Kashin-Beck disease (a type of osteoarthritis).<br>Excessive selenium exposure, whether through diet or supplements, can lead to	Regulatory agencies have established dietary reference values for selenium to help guide safe intake levels.	Selenium is an essential trace element that is crucial for human health when consumed in appropriate amounts. It plays a key role in various physiological processes, including antioxidant defense and the functioning of the thyroid gland. However, both deficiency and excessive exposure to selenium can lead to health issues.

		primarily through dietary sources, such as fish, meat, nuts, grains, and vegetables. In appropriate amounts, dietary selenium is necessary for normal growth, development, and overall health. Selenium is essential for the proper functioning of enzymes involved in protecting cells from oxidative damage and in thyroid hormone metabolism.	selenosis, a condition characterized by a range of health issues.<br>Symptoms of selenosis may include gastrointestinal problems (nausea, diarrhea), hair and nail changes (brittleness), skin rashes, and neurological symptoms (e.g., numbness).<br>Chronic overexposure to selenium may lead to more severe symptoms, such as hair and nail loss, joint pain, and even respiratory problems.		
Rb	Some industrial processes, such as the production of specialty glass, can lead to occupational exposure to rubidium. In such cases, inhalation of rubidium compounds or contact with	Rubidium is found in trace amounts in the Earth's crust and can be present in minerals, soil, and groundwater. Exposure to rubidium for the general population typically occurs through the	Rubidium is not considered toxic in small quantities, and the general population is not typically at risk of adverse health effects due to rubidium exposure.		As with many elements, individual sensitivity to rubidium can vary. Some individuals may be more sensitive to certain metals or elements and may experience adverse effects at lower exposure levels.

	rubidium-containing materials may occur.	consumption of food and water			
Sr	Strontium-90 is a radioactive isotope of strontium and is produced as a byproduct of nuclear fission. Exposure to strontium-90 is of particular concern due to its radioactive properties.<br>Strontium-90 behaves similarly to calcium in the body, which can lead to its incorporation into bone tissue, potentially increasing the risk of bone cancer and leukemia.<br>Strontium-90 can be released into the environment from nuclear testing, nuclear accidents, or nuclear waste disposal.	Small amounts of naturally occurring strontium can be found in soil, water, and food. In small quantities, strontium is not generally considered harmful and is not typically a significant source of exposure.<br>In certain regions, strontium may be present in higher concentrations in drinking water due to geological factors. Such exposure levels are usually not a cause for concern.	Non-Radioactive Strontium: In its non-radioactive form, strontium is not considered toxic at normal environmental exposure levels. In fact, non-radioactive strontium salts are sometimes used in dietary supplements to support bone health.<br>Radioactive Strontium-90: Exposure to radioactive strontium-90 is a serious health concern. It can lead to increased cancer risk, particularly bone cancer, due to the radiation emitted by this isotope. Proper disposal and containment of strontium-90 and other radioactive materials are essential to prevent exposure.	Regulatory agencies closely monitor and regulate the release and disposal of radioactive materials, including strontium-90, to minimize environmental and health risks.	exposure to non-radioactive strontium at normal environmental levels is generally considered safe and can even have potential health benefits. However, exposure to radioactive strontium-90 is a significant concern due to its potential to increase the risk of cancer, especially bone cancer. Proper containment and disposal of radioactive materials are crucial to prevent exposure and minimize associated health risks.

Mo	Workers in certain industrial settings, such as mining and metal production, may be at risk of occupational exposure to molybdenum, particularly through inhalation of molybdenum dust or fumes. Proper workplace safety measures should be followed to minimize exposure.	Molybdenum is obtained primarily through dietary sources, such as legumes, grains, leafy vegetables, and nuts. In appropriate amounts, dietary molybdenum is essential for normal growth and development. Molybdenum is a cofactor for several enzymes involved in key metabolic processes, including the breakdown of certain amino acids.	Essential Trace Element: Molybdenum is vital for human health at appropriate levels. It is not associated with acute toxicity or adverse health effects when consumed as part of a balanced diet.<br>Genetic Conditions: There are rare genetic conditions, such as molybdenum cofactor deficiency, that can result in severe health problems related to the absence of molybdenum-dependent enzymes. These conditions are typically diagnosed in infancy and require specialized medical care. While molybdenum is an essential nutrient, excessive intake through dietary supplements is not recommended. High levels of dietary molybdenum can interfere with the absorption of copper and result in copper deficiency, which can lead to health issues.	Regulatory agencies have established permissible exposure limits for molybdenum in occupational settings to protect workers from excessive exposure.	Exposure to molybdenum is generally not a health concern when obtained through a balanced diet at typical environmental levels. In fact, molybdenum is an essential trace element necessary for normal human health. It is crucial to avoid excessive intake of molybdenum through dietary supplements, which could lead to adverse health effects by interfering with copper absorption. Occupational exposure to molybdenum should be managed through safety protocols to minimize potential risks.
Ag	Workers in certain industries, such as those involved in silver mining, refining, and manufacturing of silver-containing products, may be at risk of occupational exposure to silver	Silver is naturally present in very small amounts in the environment, and it can be ingested through the diet. Silver can enter the environment through industrial	The use of colloidal silver, a suspension of tiny silver particles in a liquid, as an alternative medicine has been associated with argyria and other health risks when consumed in excessive amounts.<br>Dental amalgams that contain silver are used as dental fillings. These fillings are generally considered safe, but there has been some debate about their safety and	The FDA has issued warnings about the use of such products.	

	dust, fumes, or compounds.	discharge and waste disposal.<br>High levels of silver in water and soil can affect aquatic ecosystems.<br>Exposure through environmental sources is generally not a concern for humans.	potential exposure to mercury, which is also present in dental amalgams.		
Cd	Workers in certain industries, such as mining, smelting, and manufacturing of batteries, pigments, and plastics, may be at risk of occupational exposure to cadmium through inhalation and skin contact with cadmium dust or fumes.	Tobacco plants readily accumulate cadmium from the soil, and smoking cigarettes is a significant source of cadmium exposure for smokers and those exposed to secondhand smoke.<br>: Cadmium can enter the food chain through contaminated soil and water. Some foods, especially shellfish, grains, and vegetables, may contain	Inhalation of cadmium fumes or dust can lead to acute and chronic respiratory problems, including coughing, shortness of breath, and chronic obstructive pulmonary disease (COPD).<br>Cadmium is primarily known for its toxic effects on the kidneys. Long-term exposure can lead to kidney damage and kidney disease.<br>Cadmium can replace calcium in bones, resulting in weakened bones and increased fracture risk.<br>Cadmium is classified as a Group 1 human carcinogen by the International Agency for Research on Cancer (IARC). Prolonged exposure to cadmium is associated with an increased risk of lung, prostate, and other cancers.<br>Cadmium exposure can affect fertility and lead to adverse reproductive outcomes.	Workers in industries with potential cadmium exposure should follow safety protocols and use personal protective equipment to minimize exposure.<br>: Avoiding tobacco smoke is a significant step in reducing cadmium exposure.<br>Consuming a balanced diet with foods low in cadmium and avoiding excessive intake of contaminated foods can help reduce exposure.	

		elevated cadmium levels.<br>Some household products, such as rechargeable batteries, may contain cadmium.			
Sn	Workers in industries where tin or tin compounds are used may have potential for occupational exposure. Appropriate workplace safety measures should be followed to minimize exposure. Long-term, high-level exposure may lead to respiratory symptoms or skin irritation.	Tin is not commonly found at levels in the environment that pose health risks to humans. It is typically not a significant source of exposure.<br>Tin is found naturally in trace amounts in various foods and beverages, as well as in certain food packaging materials (e.g., tin cans). In these typical dietary exposures, tin is not considered harmful and is generally safe for human consumption.	Low Toxicity: Tin is generally considered to have low toxicity, and acute exposure to tin at normal environmental levels or through dietary intake is not typically associated with severe health effects.	Regulatory agencies have established limits on permissible levels of tin exposure in the workplace to protect workers from excessive exposure.	Exposure to tin, a metallic element, is generally not associated with significant health risks when encountered at typical environmental levels or through dietary intake. Tin is considered to be of low toxicity. Tin cans used for packaging foods typically have a layer of tin on the interior surface to prevent the can from corroding. The tin lining is considered safe for food storage and consumption.

Sb	Workers in industries that involve antimony mining, refining, or the production of antimony-containing products, such as batteries or flame retardants, may be at risk of occupational exposure to antimony. Exposure can occur through inhalation of antimony dust or fumes, as well as skin contact.	Environmental exposure to antimony is generally not a significant concern for the general population, as it is not commonly found in the environment at levels that pose health risks.	Respiratory Effects: Inhalation of antimony dust or fumes, particularly in occupational settings, can lead to respiratory issues, including coughing, chest pain, and shortness of breath.<br>Gastrointestinal Effects: Ingesting high levels of certain antimony compounds can lead to gastrointestinal distress, including nausea, vomiting, and diarrhea.<br>Skin Effects: Skin contact with antimony compounds can result in skin irritation and rashes.<br>Cardiovascular Effects: Chronic exposure to antimony compounds may lead to cardiovascular issues, such as changes in the electrical activity of the heart.	Regulatory agencies have established permissible exposure limits for antimony in the workplace to protect workers from excessive exposure.	Exposure to antimony, a naturally occurring element, can have varying health effects depending on the specific form of antimony and the level of exposure. Antimony is used in various industrial applications, and certain antimony compounds can be toxic.
Cs		Cesium-137 is a radioactive isotope of cesium that is not naturally occurring but is a product of nuclear fission, commonly found in nuclear waste. It is released during nuclear accidents, nuclear weapon tests, and from the nuclear fuel cycle.	Radiation Exposure: Cs-137 emits ionizing radiation, which can damage living tissues, particularly when it is incorporated into the body. This radiation can increase the risk of cancer, including leukemia, and damage organs and tissues.<br>Contamination: Cs-137 can contaminate soil, water, and crops, and it may enter the food chain. Individuals who consume contaminated food or water can be exposed to Cs-137.<br>Acute Effects: Acute exposure to high levels of Cs-137 can result in symptoms such as nausea, vomiting, diarrhea, and potentially fatal radiation sickness.	Regulatory agencies closely monitor and regulate the handling and disposal of radioactive materials, including Cs-137, to minimize environmental and health risks.<br>In the event of a nuclear accident or contamination, public health authorities may issue advisories and take measures to prevent further exposure and	Exposure to cesium, a metallic element, can have various health effects, particularly when the radioactive isotope cesium-137 (Cs-137) is involved.

		Cs-137 behaves chemically like non-radioactive cesium and can be absorbed by the body, primarily through the ingestion of contaminated food and water. Exposure to Cs-137 is a significant concern due to its potential for causing long-term health effects.	Chronic Effects: Chronic exposure to Cs-137 over time may increase the risk of cancer and other long-term health problems.	provide appropriate medical treatment.	
Ba	Workers in industries that involve barium mining, refining, or the production of barium-containing products, such as ceramics, glass, and chemicals, may be at risk of occupational exposure to barium. Exposure can occur through inhalation of barium dust or fumes, as well as skin contact.	Environmental exposure to barium is generally not a significant concern for the general population, as it is not commonly found in the environment at levels that pose health risks.	Respiratory Effects: Inhalation of barium dust or fumes, particularly in occupational settings, can lead to respiratory issues, including coughing, shortness of breath, and chest pain.<br>Gastrointestinal Effects: Ingesting high levels of certain barium compounds, such as barium chloride, can lead to gastrointestinal distress, including nausea, vomiting, and diarrhea.<br>Cardiovascular Effects: Barium can affect the cardiovascular system, particularly when barium enters the bloodstream. Acute exposure to high levels of soluble barium compounds can lead to heart rhythm abnormalities.	Barium is sometimes used in radiology for certain medical imaging procedures, such as barium swallow tests. The barium used in these procedures is not the same as chemically toxic barium compounds and is used in a controlled medical context.	Exposure to barium, a metallic element, can have varying health effects depending on the specific form of barium and the level of exposure. Barium is used in various industrial applications, and certain barium compounds can be toxic.

Au	Workers in industries related to gold mining, refining, and jewelry manufacturing may be at risk of occupational exposure to gold dust, fumes, or compounds.	Exposure to gold is generally considered to be of low concern for health effects because gold is an inert and biocompatible metal. Gold is a popular material for making jewelry, and most people come into contact with gold through items like rings, necklaces, and earrings.<br>Gold is used in dentistry for crowns, inlays, and other dental restorations.	Unlike certain other metals, gold is not known to have toxic properties in its pure form.<br>Allergic reactions to gold are rare, but some individuals may develop skin sensitivities or allergies to gold jewelry, particularly if the gold is alloyed with other metals like nickel.<br>Ingesting gold is generally not a health concern.		Historically, gold has been used in some medicinal treatments, but these practices have largely been replaced by more effective treatments. Gold-based medications, such as gold salts, have been used to treat autoimmune diseases like rheumatoid arthritis.
Hg	Occupational exposure can also occur in industries like mining, chlor-alkali production, and gold extraction.	Methylmercury is aform of mercury, mainly found in certain types of fish and seafood. It bioaccumulates in aquatic ecosystems, and consumption of contaminated fish is a common	Neurological Effects: Mercury is a neurotoxin and can cause a range of neurological symptoms, including memory problems, mood disorders, and tremors. High levels of exposure can lead to Minamata disease, a severe neurological disorder.<br>Reproductive and Developmental Effects: Prenatal exposure to mercury, especially methylmercury from fish consumption, can harm fetal development and lead to	Minimizing Exposure: Reducing exposure to mercury involves consuming fish and seafood with low mercury content, practicing safe handling and disposal of mercury-containing devices, and avoiding products that may contain mercury.	

		source of exposure.<br>Inorganic Mercury: Exposure to inorganic mercury can occur in various ways, including through dental amalgams (silver fillings), some cosmetics, and certain traditional medicines. Occupational exposure can also occur in industries like mining, chlor-alkali production, and gold extraction.<br>Elemental Mercury: This is the metallic form of mercury and can be found in certain devices like thermometers, barometers, and fluorescent lights. Accidental inhalation of mercury vapor	cognitive and developmental problems in children.<br>Cardiovascular Effects: Exposure to high levels of mercury has been associated with cardiovascular issues, including increased blood pressure and the risk of heart disease.<br>Renal Effects: Inorganic mercury exposure can lead to kidney damage.<br>Dental Amalgams: While dental amalgams containing mercury have been used for many years, there is ongoing debate about the safety of these fillings and the potential release of mercury vapor.<br>Gastrointestinal Effects: Ingesting high levels of mercury can lead to gastrointestinal distress.	Occupational Safety: Workers in industries with potential mercury exposure should follow safety protocols and use personal protective equipment to minimize exposure.<br>Regulatory Limits: Regulatory agencies have established limits on mercury in various products and environmental sources.<br>Mercury exposure is preventable, and public awareness, safe workplace practices, and dietary choices can help minimize the health risks associated with mercury.	

		from broken devices can lead to exposure. Mercury can enter the environment through industrial emissions and waste disposal.			
Tl	Workers in industries involving thallium, such as the production of electronic devices, photocells, and certain types of glass, may be at risk of occupational exposure through inhalation of thallium dust or skin contact.	Thallium is not commonly found in the environment at levels that pose health risks. However, it may be present in trace amounts in soil and water due to natural occurrences or industrial activities.	Acute Poisoning: High-level acute exposure to thallium can result in severe poisoning symptoms, including gastrointestinal distress, nerve damage, hair loss, and, in severe cases, it can lead to coma or death.<br>Chronic Exposure: Chronic exposure to thallium, even at lower levels, can lead to a range of health issues, including neurological symptoms, such as numbness, tingling, and muscle weakness.<br>Cancer: Thallium is not known to be a human carcinogen.	Occupational exposure to high levels of thallium should be managed through safety protocols, including the use of personal protective equipment, to minimize health risks.	Exposure to thallium is a significant health concern due to its high toxicity. Acute poisoning with thallium can be life-threatening, and chronic exposure can lead to various neurological and health issues. Preventive measures, safety protocols, and regulatory limits are crucial to reduce exposure to thallium and to protect public health. In cases of suspected thallium poisoning or exposure, immediate medical attention is essential for proper diagnosis and treatment.
Pb	Certain industries, like construction, battery manufacturing, and	Lead exposure can occur through various sources, including lead-	Health Effects in Children:<br>Neurological Damage: Lead exposure in children can result in neurological	Preventing lead exposure is crucial. Measures include:	Exposure to lead, a toxic heavy metal, can have a range of detrimental health effects,

	painting, can expose workers to lead.	based paints in older homes, contaminated soil, drinking water from lead pipes or fixtures, occupational settings (e.g., lead-related industries), and some consumer products. Some imported goods, such as toys or cosmetics, may contain lead.<br>Some traditional or folk remedies and cosmetics may contain lead.	damage, leading to learning disabilities, lower IQ, and behavioral problems.<br>Growth Impairment: Lead can affect physical growth and development in children.<br>Anemia: Lead interferes with the production of hemoglobin, which can lead to anemia.<br>Kidney Damage: Prolonged exposure can harm the kidneys.<br>Health Effects in Adults:<br>Neurological Effects: In adults, lead exposure can cause cognitive impairment, memory problems, and mood disorders.<br>Cardiovascular Effects: It may increase the risk of high blood pressure and heart disease.<br>Reproductive Effects: Lead exposure can affect fertility and lead to miscarriages.<br>Pregnant Women: Lead exposure during pregnancy can harm both the mother and the developing fetus. It can lead to preterm birth, low birth weight, developmental issues, and cognitive problems in the child.<br>Treatment: Chelation therapy is sometimes used to remove lead from the body in cases of high exposure.	Regularly checking and addressing lead hazards in homes, particularly for families with young children.<br>Using lead-safe practices during renovation or remodeling.<br>Testing drinking water for lead and using filters or replacing lead pipes and fixtures.<br>Avoiding products or cosmetics that may contain lead.<br>Ensuring safe workplace practices in industries where lead exposure is possible.	particularly when encountered at elevated levels. Lead exposure is a significant public health concern because it can affect individuals of all ages, but it is especially harmful to children.
